# Supplementary material for: Chromothripsis during telomere crisis is independent of NHEJ, and consistent with a replicative origin
Source: Genome Res. 2019 May;29(5):737–49. doi: 10.1101/gr.240705.118 (PMC6499312; doi:10.1101/gr.240705.118)
Supplement: Supplemental Material [file supp_gr.240705.118_Supplemental_file_1.zip › contigs/annotated_contigs/DB53/contig.2.DB53_length_507_mean_cov_13.6055226824.docx]

**DB53_length_507_mean_cov_13.6055226824**

```
AATGGGATAAAATACTCACAGACTATGCATCTGACAAAGGGCCGGTATCTATAATCTATGAGGAACTCTTAACAATTAAACAAAAAGCA
 >chr8:83572904-83573127 - E=1e-122
AATGAGCTCATTAAAATGTGGGCAAAGGACATGATCAGACCTTTCTCAAAGGGAGATATACAAGAAGGCAACCAACATGAAAACATGCA

CCACATCACTAATCATCAGAGAAATGCAAATCAAAACTGCA|ACAA|TGAAAGTCCTAGAATATCTTGAAAATTTTGAGCAATGACTAG
                                          >chr8:83566249-83566537 + E=9e-162
GCTTGTCTCAGTTATTAAGGAAGGATCACTGCAAATTCATAAATATTGCTTTGAATTGTTCAAATACTGCAGACCCTTGAGAAGTGCAT

CCACCAAACAAGGCTGACTGTAATGACAAGTGAGTGGGCAGAAACCATAATGCAATTAATTTACAGATTGTGTAAAAGGAACATAAACA

CCATGTTAAAATGGGTTTATCAGTTGACTTCGGTATAGGTATAGATTTTGAAAATGTTGTCAGC
```
